# Understanding the Role of Yb^3+^ in the Nd/Yb Coupled 808-nm-Responsive Upconversion

**DOI:** 10.3389/fchem.2018.00673

**Published:** 2019-01-25

**Authors:** Nan Song, Bo Zhou, Long Yan, Jinshu Huang, Qinyuan Zhang

**Affiliations:** State Key Laboratory of Luminescent Materials and Devices, Guangdong Provincial Key Laboratory of Fiber Laser Materials and Applied Techniques, Institute of Optical Communication Materials, South China University of Technology, Guangzhou, China

**Keywords:** upconversion, energy migration, interfacial energy transfer, Nd/Yb coupled system, mechanistic study

## Abstract

The realization of upconversion at 808 nm excitation has shown great advantages in advancing the broad bioapplications of lanthanide-doped nanomaterials. In an 808 nm responsive system, Nd^3+^ and Yb^3+^ are both needed where Nd^3+^ acts as a sensitizer through absorbing the excitation irradiation. However, few studies have been dedicated to the role of Yb^3+^. Here, we report a systemic investigation on the role of Yb^3+^ by designing a set of core-shell-based nanostructures. We find that energy migration over the ytterbium sublattice plays a key role in facilitating the energy transportation, and moreover, we show that the interfacial energy transfer occurring at the core-shell interface also has a contribution to the upconversion. By optimizing the dopant concentration and surface anchoring the infrared indocyanine green dye, the 808 nm responsive upconversion is markedly enhanced. These results present an in-depth understanding of the fundamental interactions among lanthanides, and more importantly, they offer new possibilities to tune and control the upconversion of lanthanide-based luminescent materials.

## Introduction

Recently, substantial attention has been devoted to the lanthanide-doped nanoparticles due to their great infrared-to-visible photon upconversion performance (Auzel, 2004; Haase and Schafer, 2011; Zhou et al., 2015), which shows potential applications ranging from bioimaging (Zhu et al., 2017) to photodynamic therapy (Xu et al., 2017), 3D display (Deng et al., 2015), security (Lu et al., 2014), anti-counterfeiting (Li et al., 2016), and super-resolution nanoscopy (Liu et al., 2017). The unique 4f electronic configuration with abundant energy levels allows us to easily realize multi-wavelength upconversion emission bands upon infrared excitations (Chen et al., 2014; Dong et al., 2015; Zheng et al., 2015). By taking advantage of strategies including development of new host materials (Lei et al., 2017), control of local structure (Fischer et al., 2016), mechanistic exploration of new pathways using energy migration (Wang et al., 2011; Chen et al., 2017), cross-relaxation (Liu et al., 2017) and interfacial energy transfer (Zhou et al., 2016, 2018), efficient upconversion from a set of lanthanides such as Er^3+^, Tm^3+^, Ho^3+^, Tb^3+^, and Eu^3+^ was obtained. However, the most used upconversion systems are based on a Yb-sensitized design with 980 nm excitation, which makes the upconversion nanoparticles unsuitable for biological application because of the strong absorption of water at this wavelength region (Weissleder, 2001; Kobayashi et al., 2011; Zhu et al., 2017). Therefore, developing new classes of upconversion materials would be of great importance for their biomedical applications.

**Figure 1:**
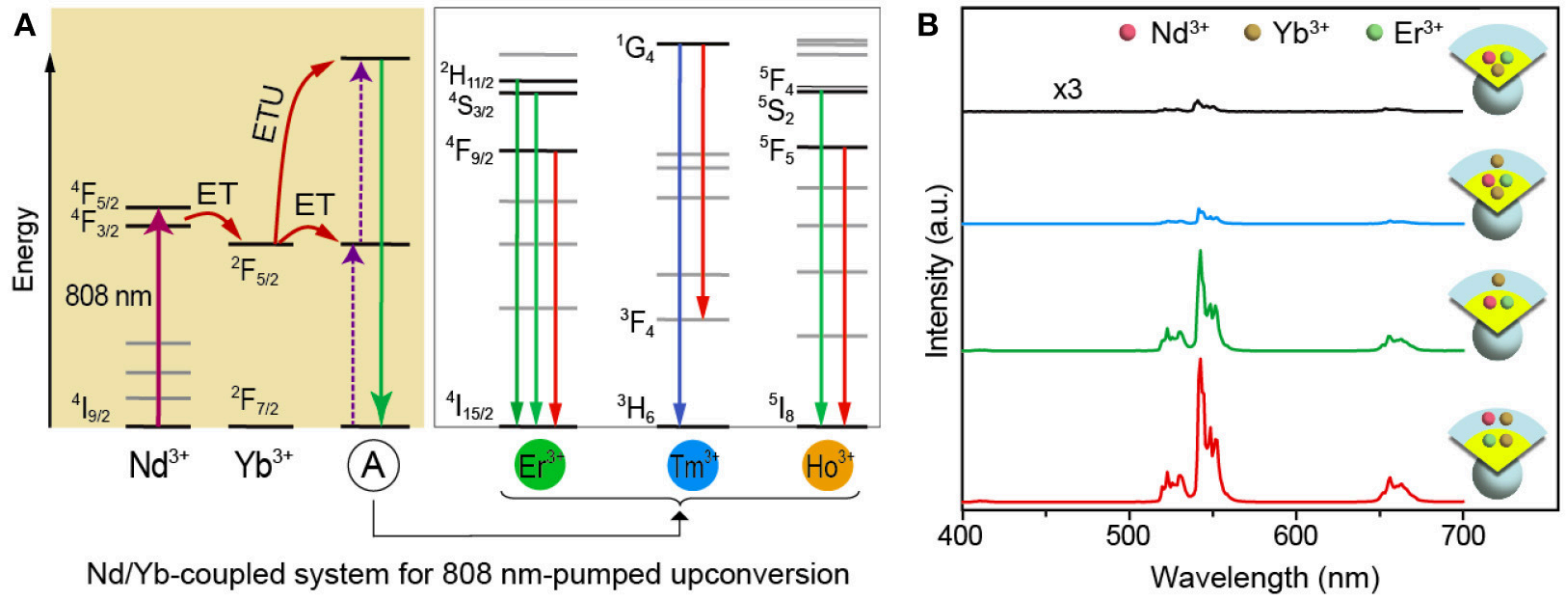
**(A)** Schematic illustration of energy transportation processes involving the Nd/Yb coupled upconversion system. The typical upconverted visible transitions from Er^3+^, Tm^3+^, and Ho^3+^ are marked in the right panel. ET and ETU stand for energy transfer and energy transfer upconversion, respectively. **(B)** Upconversion emission spectra from the four types of core-shell samples of NaYF_4_:Yb/Er/Nd(20/2/1 mol%)@NaYF_4_, NaYF_4_:Yb/Er/Nd(20/2/1 mol%)@NaYF_4_:Nd(30 mol%), NaYF_4_:Yb/Er(20/2 mol%)@NaYF_4_:Nd(30 mol%), and NaYF_4_:Yb/Er(20/2 mol%)@NaYF_4_:Nd/Yb(30/10 mol%) under 808 nm excitation.

Interestingly, recent works suggest that Nd^3+^ is a possible sensitizer to move the excitation wavelength from 980 to 808 nm due to its ^4^F_5/2_ ← ^4^I_9/2_ absorption transition at this wavelength region (Liu et al., 2017; Xie et al., 2017) together with efficient energy transfer from Nd^3+^ to Yb^3+^ (Parent et al., 1986). More importantly, the absorption of excitation energy by water in biological tissues can be effectively minimized. To date, the 808 nm pumped upconversion from a series of lanthanides (e.g., Er and Tb) has been realized (Zhong et al., 2014; Zhou et al., 2018). In the Nd-sensitized upconversion system, Yb^3+^ is also employed to facilitate the energy transfer from Nd^3+^ to the lanthanide emitter, and the typical processes involved in this system can be schematically illustrated in Figure 1A. So far, there are four typical core-shell schemes can be used to obtain the upconversion, and typical sample forms for Er^3+^ are NaYF_4_:Yb/Er/Nd(20/2/1 mol%)@NaYF_4_, NaYF_4_:Yb/Er/Nd(20/2/1 mol%)@NaYF_4_:Nd(30 mol%), NaYF_4_:Yb/Er@NaYF_4_:Nd(30 mol%), and NaYF_4_:Yb/Er(20/2 mol%)@NaYF_4_:Nd/Yb(30/10 mol%), among which the last one shows the best upconversion behavior (Figure 1B). However, few studies have explored the role of Yb^3+^, which is necessary in the 808 nm pumped upconversion systems, and the mechanism of the luminescence physics occurring in the Nd/Yb coupled upconversion is still not clear.

In this study, we performed a mechanistic investigation on the role of Yb^3+^ in the Nd/Yb coupled 808 nm responsive upconversion. We demonstrated that energy migration over the Yb-sublattice plays a key role in facilitating the energy transportation from the Nd^3+^ sensitizer to the lanthanide emitter. More importantly, we showed that the interfacial energy transfer from Yb^3+^ in the shell to the lanthanide emitter in the core across the core-shell interface also contributes to the upconversion. Such an 808 nm responsive upconversion can be markedly enhanced by optimizing the sample structure together with surface anchoring the infrared indocyanine green (ICG) dye. Our results present an in-depth understanding of the luminescence mechanism involving the Nd/Yb coupled upconversion nanomaterials, which would contribute to both fundamental research and practical applications of lanthanide-doped luminescent materials.

## Experimental

### Materials

The materials including yttrium(III) acetate hydrate (99.9%), erbium(III) acetate hydrate (99.9%), ytterbium(III) acetate hydrate (99.99%), thulium(III) acetate hydrate (99.9%), holmium(III) acetate hydrate (99.9%), neodymium(III) acetate hydrate (99.9%), oleic acid (90%), 1-octadecene (90%), sodium hydroxide (NaOH; >98%), and ammonium fluoride (NH_4_F; >98%) were all purchased from *Sigma-Aldrich*. The nitrosonium tetrafluoroborate (NOBF_4_; 98%) was purchased from *Alfa Aesar* and indocyanine green (C_43_H_47_N_2_NaO_6_S_2_), N,N-dimethylformamide (DMF; anhydrous, 99.8%) were purchased from *Energy Chemical*. All these materials were used as received unless otherwise noted.

### Sample Synthesis

The core-shell based nanoparticles were synthesized using a coprecipitation chemical method, which was shown to be a good way for the preparation of core only and core-shell based nanoparticles (Wang et al., 2011). The core nanoparticle samples were pre-synthesized as the seeds for growth of the core-shell structure. In a typical procedure for the synthesis of NaYF_4_:Yb/Er core nanoparticles, to a 50-mL flask containing oleic acid (3 mL) and 1-octadecene (7 mL) was added a water solution containing Y(CH_3_CO_2_)_3_, Yb(CH_3_CO_2_)_3_, and Er(CH_3_CO_2_)_3_ at designed ratios (e.g., 78:20:2 mol%) with a total amount of 0.4 mmol. The resulting mixture was heated at 150°C for 1 h and then cooled down to room temperature. Subsequently, a methanol solution containing NaOH (1 mmol) and NH_4_F (1.6 mmol) was added and stirred at 50°C for 0.5 h, and then heated at 290°C under an argon flow for 1.5 h before cooling down to room temperature. The resulting core nanoparticles were collected by centrifugation, washed with ethanol, and finally dispersed in cyclohexane. Other control core nanoparticles were synthesized using a similar procedure except for the use of different lanthanide precursors.

Next, the core-shell nanoparticles were prepared with a two-step coprecipitation method using the pre-synthesized core nanoparticles as seeds for shell layer growth. Typically, for the synthesis of NaYF_4_:Yb/Er@NaYF_4_:Nd/Yb core-shell nanoparticles, to a 50-mL flask containing oleic acid (3 mL) and 1-octadecene (7 mL) was added a water solution containing Y(CH_3_CO_2_)_3_, Nd(CH_3_CO_2_)_3_, and Yb(CH_3_CO_2_)_3_ at designed ratios (e.g., 40:50:10 mol%) with a total amount of 0.4 mmol. The resulting mixture was heated at 150°C for 1 h and then cooled down to room temperature. Subsequently, the pre-synthesized NaYF_4_:Yb/Er particles were added as seeds along with a methanol solution containing NaOH (1 mmol) and NH_4_F (1.6 mmol) was added and stirred at 50°C for 0.5 h, and then heated at 290°C under an argon flow for 1.5 h before cooling down to room temperature. The resulting nanoparticles were collected by centrifugation, washed with ethanol, and finally dispersed in cyclohexane. The core-shell-shell nanoparticles were prepared through a three-step coprecipitation method with a similar procedure except for using pre-synthesized core-shell particles as the seeds for the outermost shell layer growth.

The following synthetic procedure was used to prepare dye-decorated nanoparticles. Firstly, the sub-nanometer ligands of NOBF_4_ were used to exchange the oleic acid ligands for the nanoparticles by coprecipatition method. The nanoparticles despersed in cyclohexane were mixed with the DMF solution of NOBF_4_ (0.1 M) at room temperature, and the mixture was shaken gently for minutes to extract nanoparticles from upper cyclohexane layer into the bottom DMF layer. The bottom layer solution was then centrifuged at 11,000 rpm for 25 min, and the precipitated nanoparticles were weighted and re-dispersed in DMF (~60 mg/mL) for NIR dye sensitization experiment. Subsequently, a suitable amount of the ICG dyes dissolved DMF solution (1 μg/mL) was added to the nanoparticle dispersed DMF solution. This mixture was stirred overnight at room temperature to produce the ICG dyes sensitized nanoparticles in DMF solution.

### Characterization

The powder X-ray diffraction (XRD) data were recorded on a Philips Model PW1830 X-ray powder diffractometer with Cu Kα radiation (λ = 1.5406 Å). The upconversion emission spectra and infrared emission spectra were measured by a Jobin-Yvon Triax 320 spectrofluorometer equipped with an 808-nm laser diode with power density of 27.9 W/cm^2^. A 980-nm laser diode with identical power density was also used for the excitation of control samples. The decay curves were measured with the same spectrofluorometer using the pulsed lasers as excitation sources. The low- and high-resolution transmission electron microscopy (TEM) measurements, together with elements mappings, were performed on a JEM 2100F with an acceleration voltage of 200 kV. The upconversion emission photographs were taken with a digital camera.

## Results and Discussion

**Figure 2:**
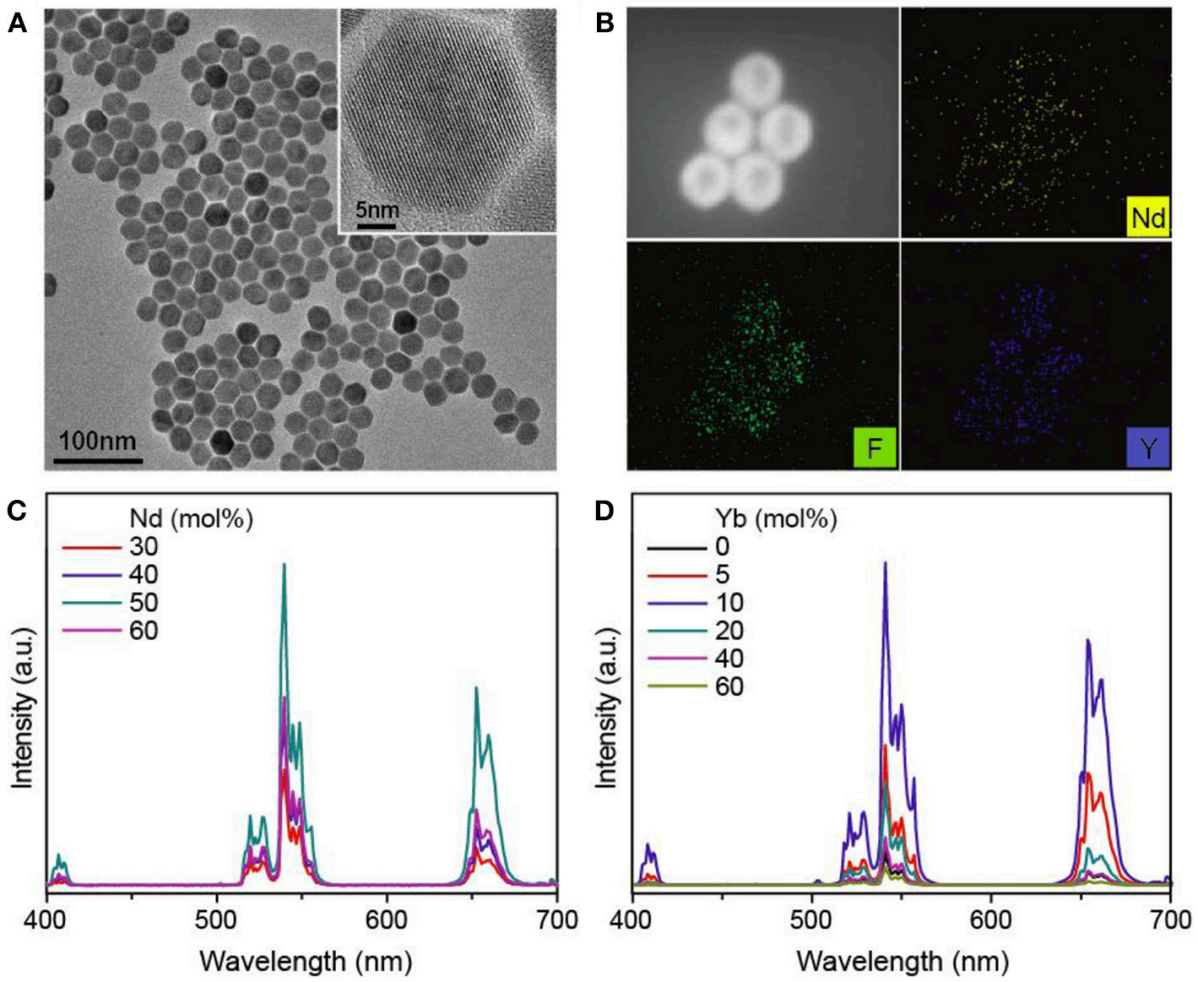
**(A)** TEM image of the as-synthesized NaYF_4_:Yb/Er(20/2 mol%)@NaYF_4_:Nd/Yb(50/10 mol%) core-shell nanoparticles. Inset shows the high-resolution TEM image of a single nanoparticle. **(B)** STEM image of the **(A)** sample and the element mappings of Y, Nd, and F. **(C,D)** Upconversion emission spectra from **(C)** NaYF_4_:Yb/Er(20/2 mol%)@NaYF_4_:Nd/Yb(x/10; *x* = 30~60 mol%) and **(D)** NaYF_4_:Yb/Er(20/2 mol%)@NaYF_4_:Nd/Yb(40/y; *y* = 0~60 mol%) core-shell nanoparticles under 808 nm excitation.

We firstly made an optimization of the upconversion from the NaYF_4_:Yb/Er@NaYF_4_:Nd/Yb core-shell nanoparticles at 808 nm excitation. Here the typical NaYF_4_:Yb/Er(20/2 mol%) nanoparticles were used as core seeds because of their good upconverted luminescence (Figure S1). The as-synthesized core-shell nanoparticles showed good monodispersive characteristic (Figures 2A,B) and are in hexagonal phase according to the XRD diffraction profile (Figure S2). As shown in Figure 2C, the increment of Nd^3+^ concentration in the shell layer contributes to an enhancement of the visible upconversion of Er^3+^ and the optimal concentration of Nd^3+^ is found to be 50 mol%. It is easily understood that heavier dopant concentration of sensitizer can result in a higher absorption of the incident excitation irradiation. The upconversion as a function of Yb^3+^ concentration in the shell was also investigated, and the result is shown in Figure 2D. The presence of Yb^3+^ in the shell layer indeed leads to the enhancement of upconversion; however, a higher dopant concentration can cause a serious luminescence quenching, and a concentration of 10 mol% is found to be the optimized value with 12.3 times enhancement (Table S1). Thus, the optimal concentration of dopants in the shell layer for Nd-Yb pair is determined to be 50 and 10 mol%, respectively. The upconversion performance from Tm^3+^ and Ho^3+^ was also measured by preparing the NaYF_4_:Yb/A(A=Tm,Ho)@NaYF_4_:Nd/Yb core-shell samples, and typical upconversion emission profiles were obtained (Figure S3). Because Er^3+^ exhibits the most intensive upconversion emission, it was used in the following experiments.

**Figure 3:**
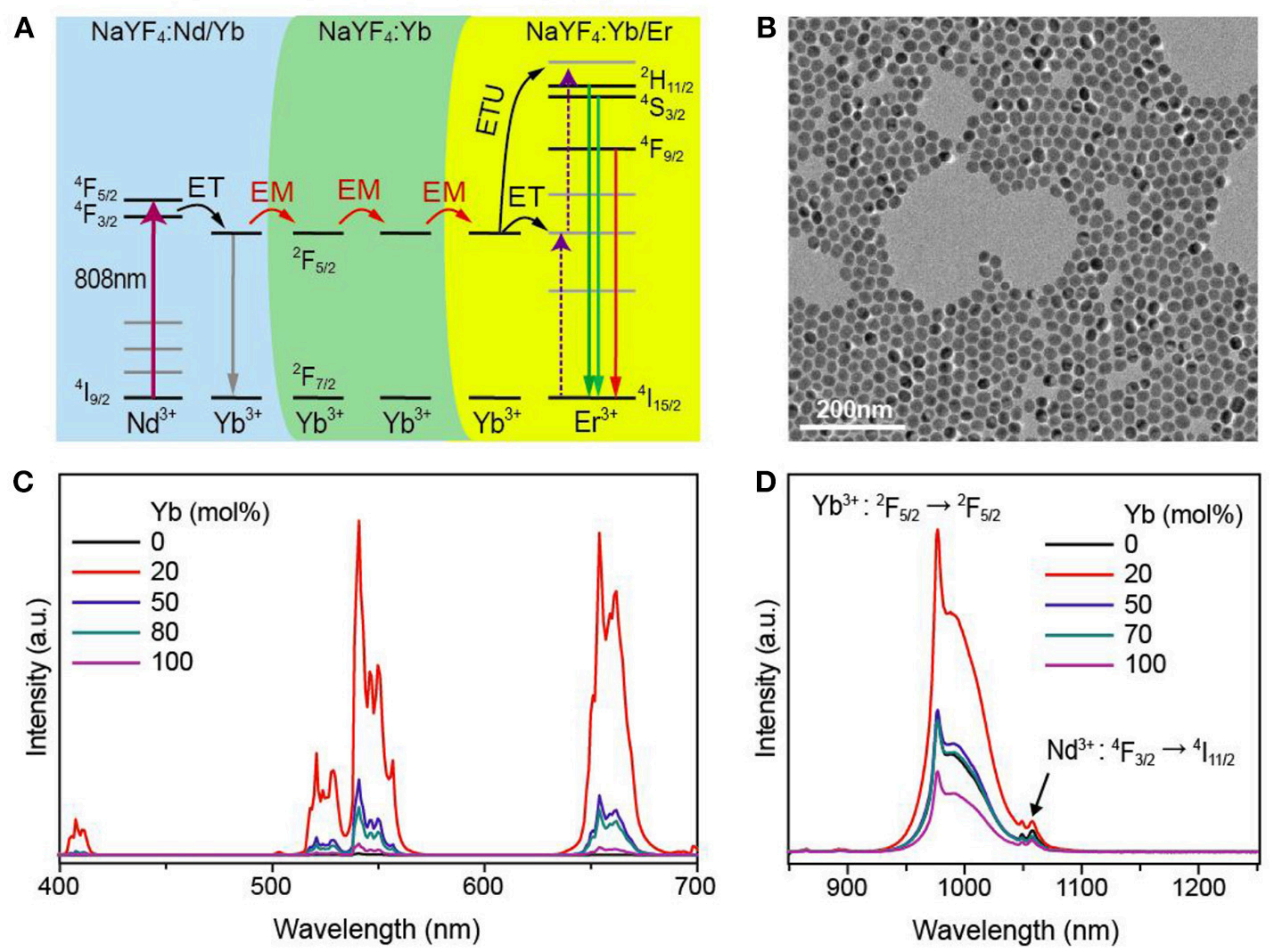
**(A)** Schematic of proposed NaYF_4_:Yb/Er@NaYF_4_:Yb@NaYF_4_:Nd/Yb core-shell-shell nanostructure for investigating the role of energy migration involving Yb-sublattice. **(B)** TEM image of the as-synthesized NaYF_4_:Yb/Er(20/2 mol%)@NaYF_4_:Yb(20 mol%)@NaYF_4_:Nd/Yb(50/10 mol%) core-shell-shell nanoparticles. **(C)** Upconversion emission spectra from the **(A)** samples with a fine tuning of Yb^3+^ concentration under 808 nm excitation. **(D)** Near infrared emission spectra from **(C)** samples under 808 nm excitation.

In order to investigate the possible energy migration involving the Nd/Yb coupled upconversion system, we propose a core-shell-shell trilayer nanostructure by inserting the Yb-doped interlayer into the NaYF_4_:Yb/Er(20/2 mol%)@NaYF_4_:Nd/Yb(50/10 mol%) core-shell nanostructure, as schematically shown in Figure 3A. In this case, the upconversion of Er^3+^ from the core should be closely dependent on the content of Yb^3+^ in the interlayer since it acts as a bridge to facilitate the energy transportation from the outermost shell layer to the core under 808 nm irradiation. These nanoparticles were successfully prepared using the three-step co-precipitation method (Figure 3B) and their upconversion emission spectra are shown in Figure 3C. It is clearly observed that the upconverted emission from Er^3+^ produces an initial increase and then a decline with the increase of Yb^3+^ content. More importantly, almost no Er^3+^ upconversion is observed for the sample without Yb^3+^ doping in the interlayer. These results clearly confirmed the occurrence of energy migration among Yb-sublattice, and the optimal Yb^3+^ content is at around 20 mol%. On the other hand, intense infrared emission bands of Yb^3+^ were also recorded, see Figure 3D. This indicates that the spontaneous ^2^F_5/2_→^2^F_7/2_ transition is also a leading de-excitation channel for Yb^3+^ ions apart from energy migration.

**Figure 4:**
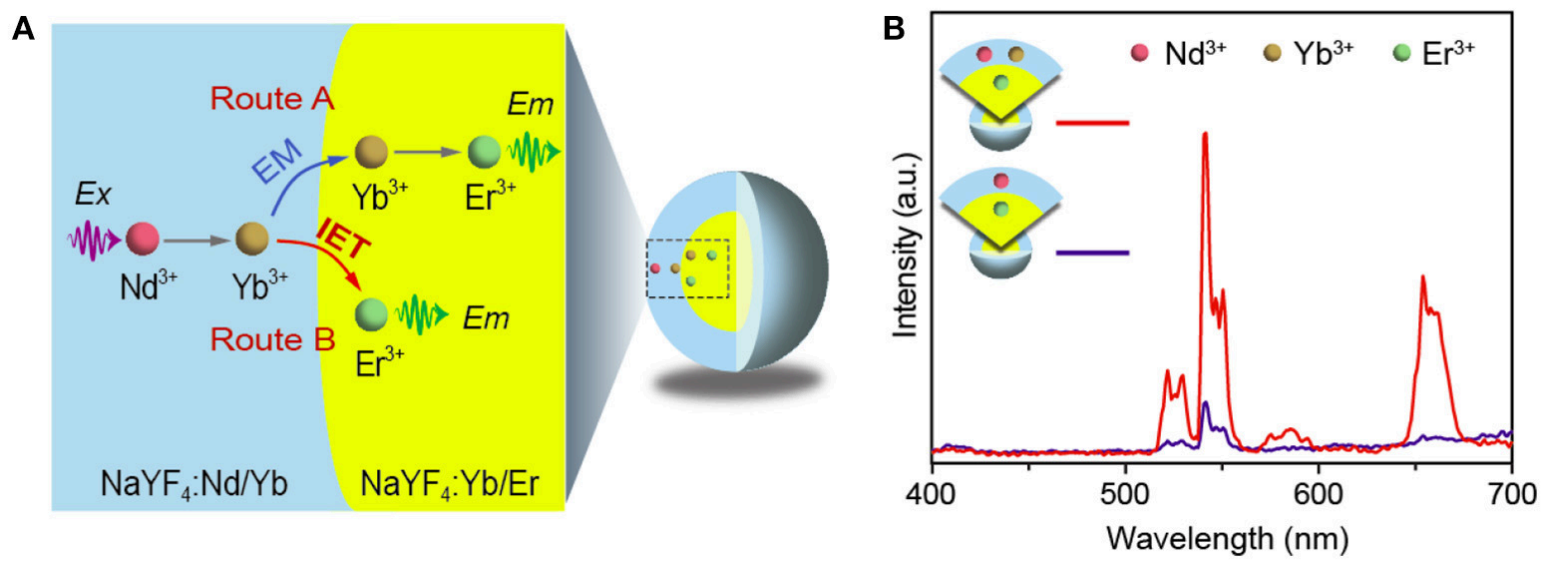
**(A)** Schematic of possible energy migration (EM; Route A) and interfacial energy transfer (IET; Route B) in the design of NaYF_4_:Yb/Er@NaYF_4_:Nd/Yb core-shell nanostructure. Ex and Em stand for excitation and emission, respectively. **(B)** Upconversion emission spectra from control samples of NaYF_4_:Er(2 mol%)@NaYF_4_:Nd/Yb(50/10 mol%) and NaYF_4_:Er(2 mol%)@NaYF_4_:Nd(50 mol%) under 808 nm excitation.

We recently discovered that Yb-mediated interfacial energy transfer is an efficient process for enabling the upconversion from lanthanides (Zhou et al., 2018). Thus, there might exist a channel to activate the lanthanide emitter through a way of interfacial energy transfer in addition to the energy migration involving Yb-sublattice, as schematically illustrated in Figure 4A. Then two control core-shell samples of NaYF_4_:Er@NaYF_4_:Nd/Yb and NaYF_4_:Er@NaYF_4_:Nd were synthesized to check the role of Yb^3+^ in the shell layer. Note that no Yb^3+^ was incorporated into the core aiming to remove the possible interference of Yb^3+^ from the core on the resultant upconversion. Interestingly, the upconverted emission of Er^3+^ was markedly enhanced for the core-shell sample after the presence of Yb^3+^ in the shell layer (Figure 4B). This observation confirmed that the Yb^3+^ in the shell layer plays a key role in transporting the excitation energy from the shell layer to the core, and more importantly, it verified that the interfacial energy transfer from the Yb^3+^ in the shell to the Er^3+^ in the core indeed occur. Therefore, it can be concluded that both processes of energy migration and interfacial energy transfer contribute to the observation of efficient upconversion from the Nd/Yb coupled nanosystem.

**Figure 5:**
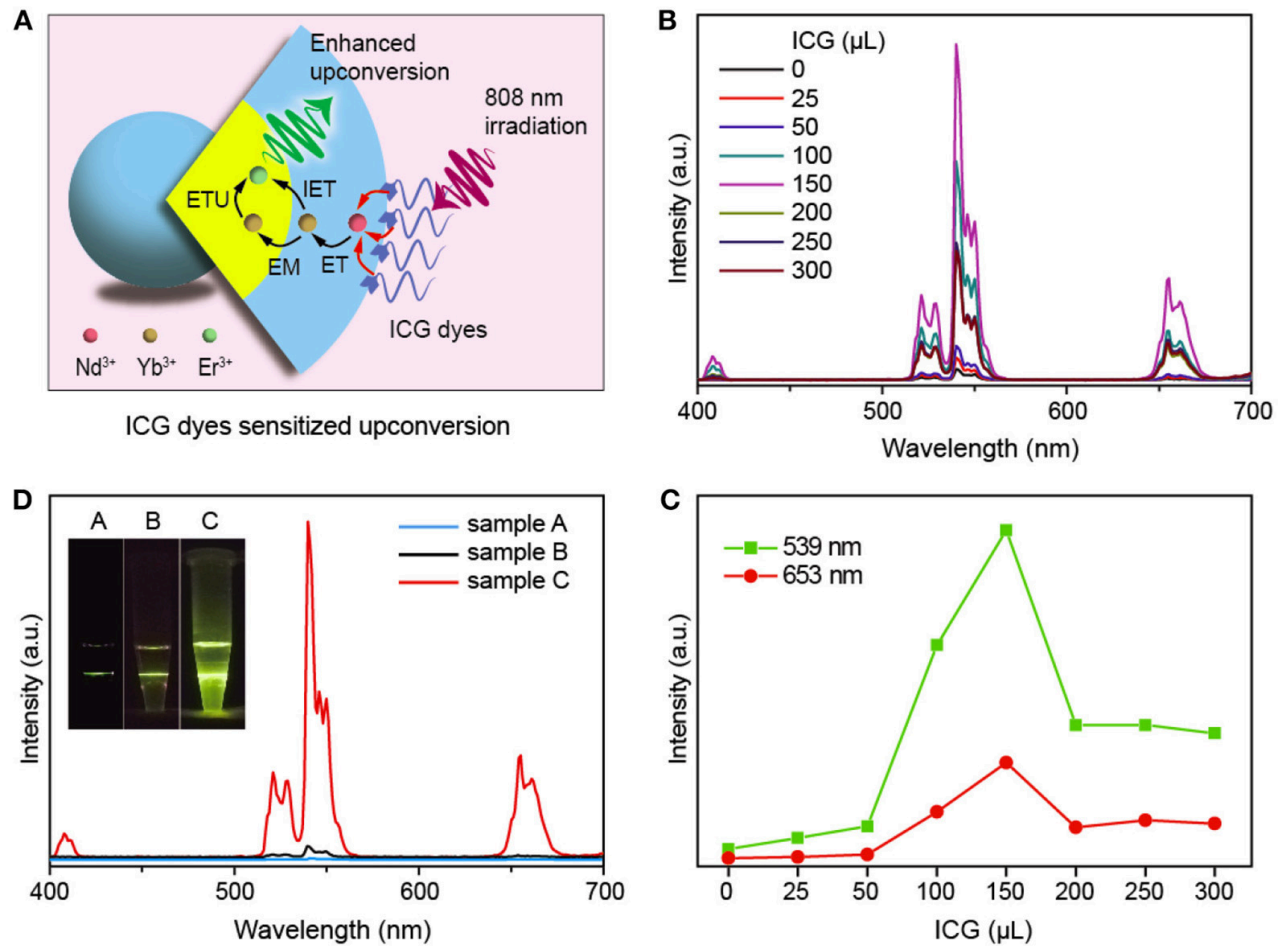
**(A)** Schematic of enhancing upconversion through using the infrared ICG dye. **(B)** Upconversion emission spectra from the **(B)** NaYF_4_:Yb/Er(20/2 mol%)@NaYF_4_:Nd/Yb(50/10 mol%) core-shell nanoparticles sensitized with different amount of ICG dyes (1 μg/mL) under 808 nm excitation. **(C)** Dependence of upconversion emission intensity on the content of ICG. **(D)** A comparison of the upconversion from the NaYF_4_:Yb/Er/Nd(20/2/1 mol%)@NaYF_4_:Nd(30 mol%) (sample A) and NaYF_4_:Yb/Er(20/2 mol%)@NaYF_4_:Nd/Yb(50/10 mol%) (sample B), and the ICG dye sensitized NaYF_4_:Yb/Er(20/2 mol%)@NaYF_4_:Nd/Yb(50/10 mol%) (sample C) core-shell-shell nanoparticles. Inset shows their emission photographs under 808 nm irradiation.

Such an 808 nm enabled upconversion allows for a further enhancement of the upconversion by introducing the infrared dyes which have much higher absorption ability than Nd^3+^ at 808 nm wavelength region (Figure 5A), and the subsequent energy transfer from dye to Nd^3+^ could help greatly enhance the upconversion in this system (Zou et al., 2012; Wu et al., 2016; Wang et al., 2017). Considering the high absorption cross section (~6 × 10^−16^ cm^2^) of indocyanine green (ICG) dye which is ~5,000 times higher than that of Nd^3+^ (1.2 × 10^−19^ cm^2^) at around 800 nm (Kushida et al., 1968; De Boni and Mendonca, 2011), here we used it to sensitize the upconversion from the present NaYF_4_:Yb/Er@NaYF_4_:Nd/Yb core-shell nanoparticles (Wang et al., 2018). As shown in Figures 5B,C, the emission intensity is markedly enhanced when using the ICG dye amount of 150 μL (1 μg/mL), confirming the effectiveness of the construction in Figure 5A. The detail of energy transportation was shown in Figure S4. It should be pointed out that this dye-sensitized upconversion is 210 times more enhanced than that from NaYF_4_:Yb/Er/Nd@NaYF_4_:Nd core-shell nanoparticles (Figure 5D) when ICG used is 150 μL, at which the number of ICG per particle is estimated to be 32.8 given a full attachment of them at the surface. This result would greatly contribute to the diversity of frontier biological applications.

**Figure 6:**
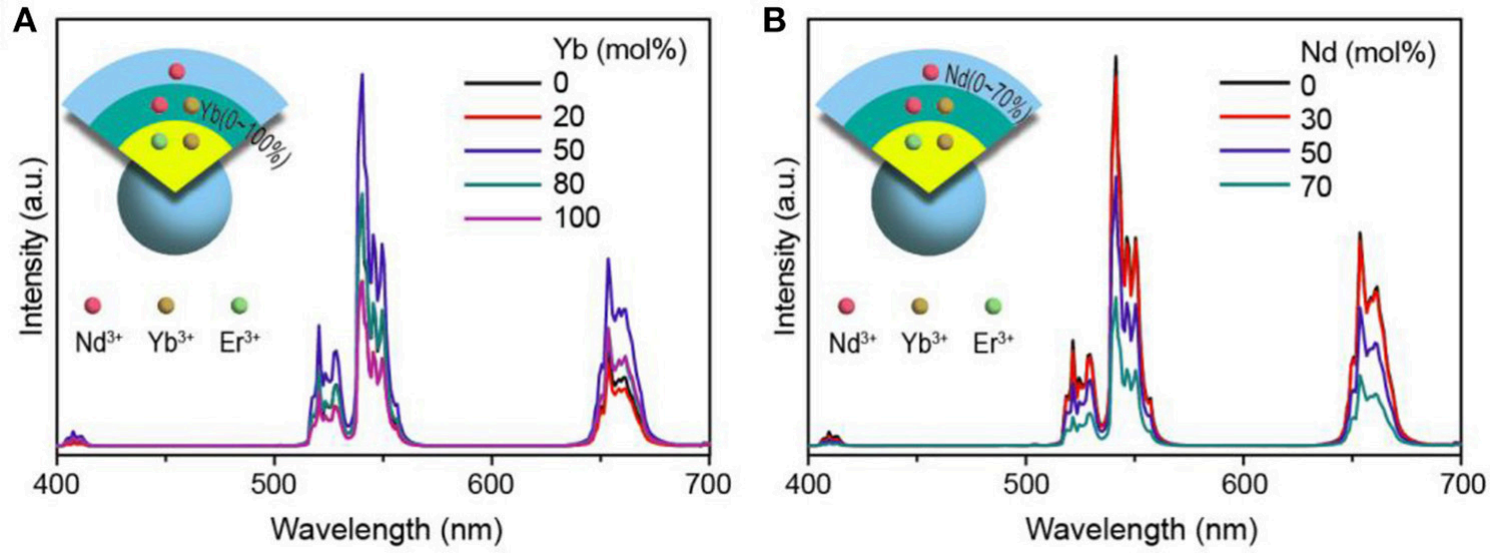
Upconversion emission spectra from the **(A)** NaYF_4_:Yb/Er(20/2 mol%)@NaNdF_4_:Yb(0~100 mol%)@NaYF_4_:Nd(50 mol%) and **(B)** NaYF_4_:Yb/Er(20/2 mol%)@NaNdF_4_:Yb(50 mol%)@NaYF_4_:Nd(0~70 mol%) core-shell-shell samples under 808 nm excitation. Insets show the structure of the trilayer samples.

On the other hand, recent studies showed that a control of energy migration involving Yb-sublattice present a novel and efficient approach to tuning and enhancing upconversion performance of lanthanides (Chen et al., 2016; Liu et al., 2018). In the present work, the presence of Yb^3+^ into the shell layer indeed leads to a decline of the upconversion (Figure S5). We then designed a NaYF_4_:Yb/Er@NaNdF_4_:Yb@NaYF_4_:Nd core-shell-shell nanostructure to improve the upconversion at 808 nm irradiation by making a fine tuning of the Yb^3+^ concentration in the Nd-sublattice (Figure 6A). It was found that 50 mol% Yb^3+^ in the NaNdF_4_ interlayer is the optimal value for balancing the absorption and further transportation of the incident 808 nm excitation energy. Note that this Yb^3+^ content (50 mol%) in the interlayer is much higher than that from the NaYF_4_:Yb/Er@NaYF_4_:Nd/Yb core-shell nanostructure (10 mol%), revealing that there might exist a possibility of energy migration over to the surface which can quench the upconversion. In this case, the designs with high doping of migratory lanthanides need an optically inert shell layer to isolate the interactions between lanthanide emitter and surface quencher (Liu et al., 2018; Yan et al., 2018). Notably, the upconversion emission intensity is much weaker for these samples without the outermost shell layer (Figure S6). We further investigated the role of Nd^3+^ in the outermost shell layer. And the spectral result shows that a doping of it in the shell layer caused a slight decline in the upconversion intensity (Figure 6B) and lifetime (Figure S7). It should be noted that the near infrared emission from Yb^3+^ can be well improved (Figure S8).

## Conclusions

In conclusion, the role of Yb^3+^ in the Nd/Yb coupled upconversion system was mechanistically investigated. By designing a NaYF_4_:Yb/Er@NaYF_4_:Yb@NaYF_4_:Nd/Yb core-shell-shell nanostructure with a Yb^3+^ content tuneable interlayer, we have confirmed that the energy migration over the ytterbium sublattice plays a critical role in facilitating the energy transportation from the sensitizer in the shell to the lanthanide emitter in the core. Interestingly, the direct interfacial energy transfer from Yb^3+^ in the shell to the emitter in the core also contributes to the upconversion. By further sensitization through using the infrared dyes, the upconversion luminescence intensity was markedly enhanced. These results on dynamics in the 808 nm pumped upconversion systems provide an in-depth mechanistic understanding of the energy interactions occurred in lanthanides, and more importantly, the markedly enhanced upconversion performance shows great promise in the diversity of biological applications.

## Author Contributions

BZ conceived and designed the experiments. NS, LY, and JH performed the experiments. BZ and QZ supervised the project. BZ wrote the manuscript with input from all authors.

### Conflict of Interest Statement

The authors declare that the research was conducted in the absence of any commercial or financial relationships that could be construed as a potential conflict of interest.
