# Supplementary material for: Understanding the Role of Yb3+ in the Nd/Yb Coupled 808-nm-Responsive Upconversion
Source: Front Chem. 2019 Jan 25;6:673. doi: 10.3389/fchem.2018.00673 (PMC6355672; doi:10.3389/fchem.2018.00673)
Supplement: Supplementary file 1 [file Table_1.DOC]

**Supporting Information**

**Understanding the role of Yb^3+^ in the Nd/Yb coupled 808-nm-responsive upconversion**

Nan Song, Bo Zhou*, Long Yan, Jinshu Huang, and Qinyuan Zhang*

*State Key Laboratory of Luminescent Materials and Devices, Guangdong Provincial Key Laboratory of Fiber Laser Materials and Applied Techniques, and Institute of Optical Communication Materials, South China University of Technology, Guangzhou, 510641, China.*

E-mails: (B.Z.) zhoubo@scut.edu.cn; (Q.Z.) qyzhang@scut.edu.cn

**Table S1**

**Table S1.** Enhancement factors for the NaYF_4_:Yb/Er(20/2 mol%)@NaYF_4_:Nd/Yb(40/y; y=0~60 mol%) sample with different Yb concentration.

| Yb concentration | 0 | 5 mol% | 10 mol% | 20 mol% | 40 mol% | 60 mol% |
| --- | --- | --- | --- | --- | --- | --- |
| Enhancement factor at 540 nm | 1.0 | 4.1 | 9.5 | 3.1 | 1.4 | 0.5 |
| Enhancement factor at 653 nm | 1.0 | 9.2 | 20.1 | 3.0 | 1.1 | 0.3 |
| Enhancement factor (total) | 1.0 | 5.5 | 12.3 | 3.1 | 1.3 | 0.5 |

**Figures S1~S8**


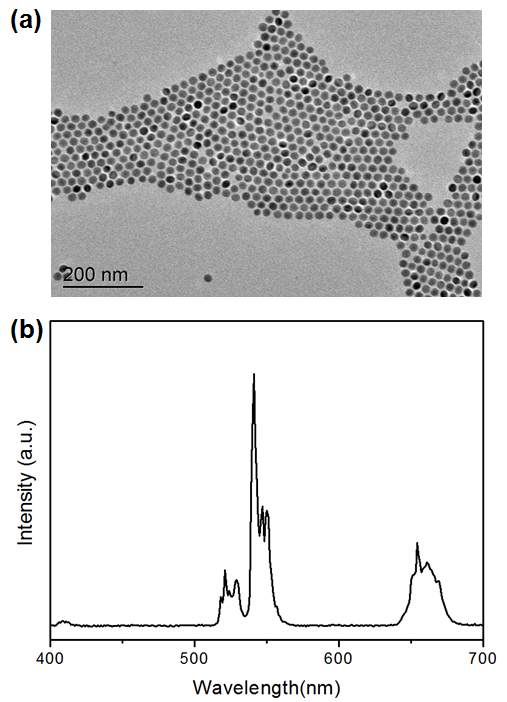


**Figure S1.** (a) TEM image of NaYF_4_:Yb/Er(20/2 mol%) nanoparticles. (b) Upconversion emission spectrum from (a) sample under 980 nm excitation.

**
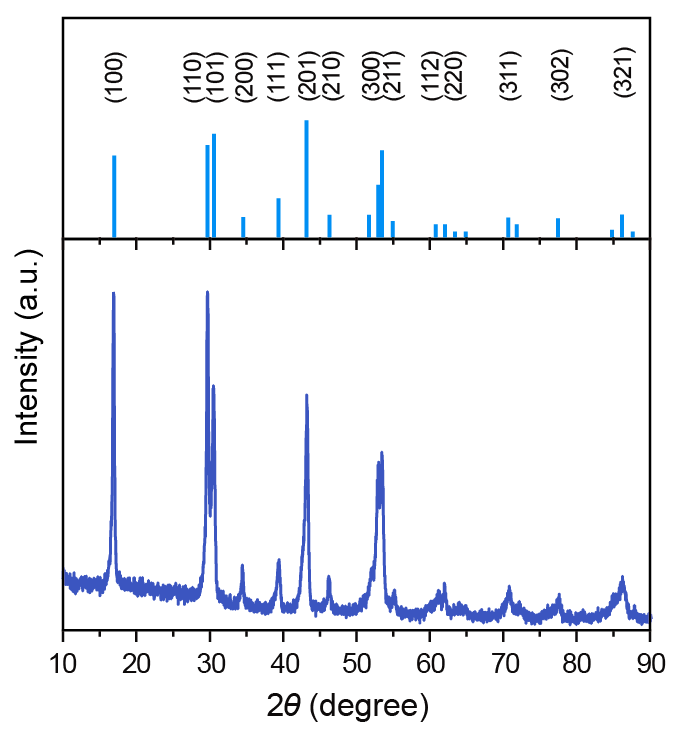
**

**Figure S2.** XRD pattern of as-synthesized NaYF_4_:Yb/Er(20/2 mol%)@NaYF_4_:Nd/Yb (50/10 mol%) core-shell nanoparticles. The card JCPDS 16-0334 from hexagonal phase NaYF_4_ is plotted for comparison.


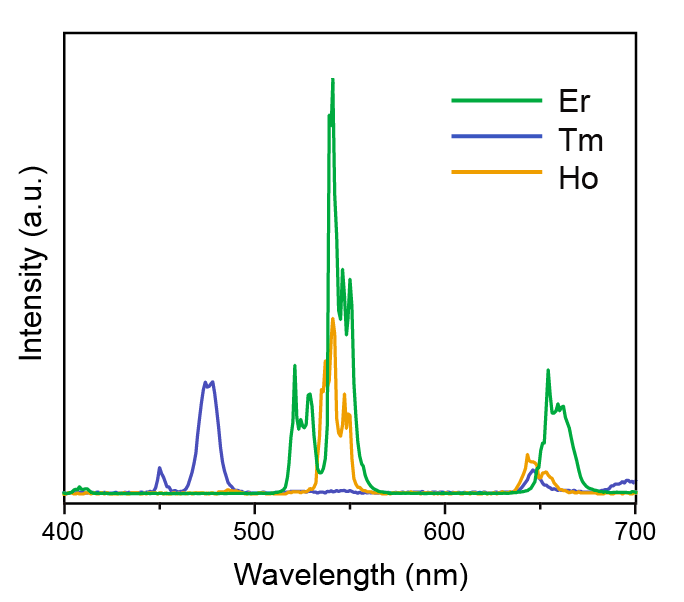


**Figure S3.** A comparison of upconversion emission spectra from NaYF_4_:Yb/Er(20/2 mol%)@NaYF_4_:Nd/Yb (50/10 mol%), NaYF_4_:Yb/Tm(30/0.5 mol%)@NaYF_4_:Nd/Yb (50/10 mol%) and NaYF_4_:Yb/Ho(20/2 mol%)@NaYF_4_:Nd/Yb (50/10 mol%) core-shell nanoparticles under identical 808 nm excitation power densities (27.9 W/cm^2^).


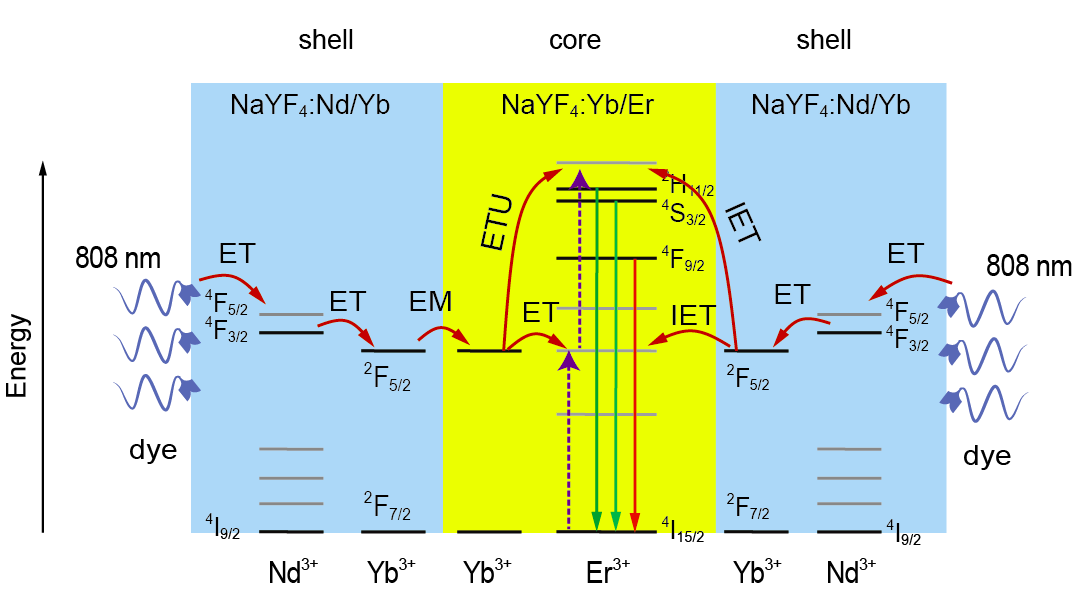


**Figure S4.** Energy transportation processes in the upconversion from the ICG dye-sensitized NaYF_4_:Yb/Er(20/2 mol%)@NaYF_4_:Nd/Yb (50/10 mol%) core-shell nanoparticles under 808 nm excitation.

**
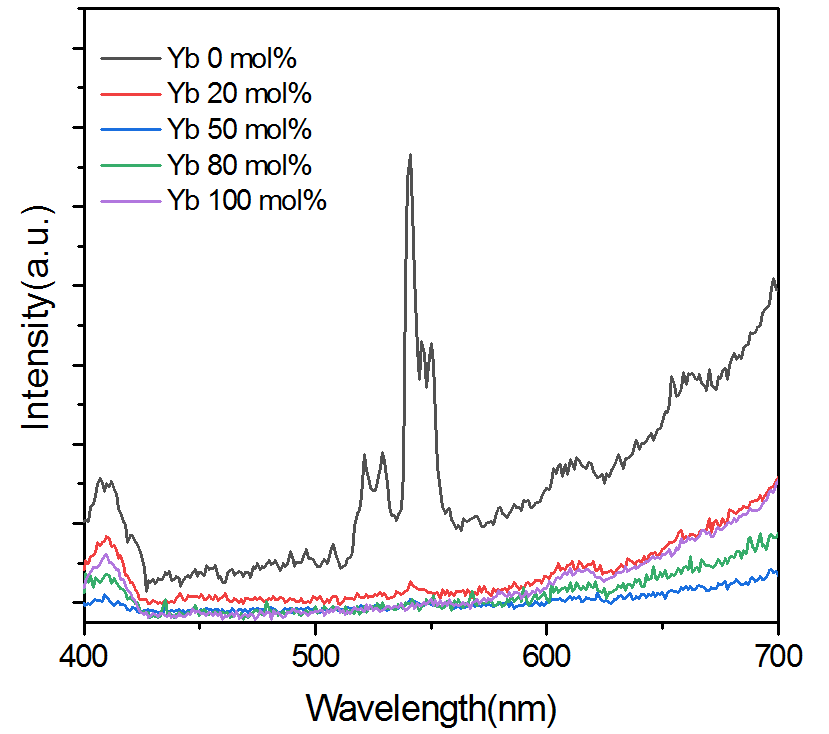
**

**Figure S5.** Upconversion emission spectra from NaYF_4_:Yb/Er(20/2 mol%)@NaYF_4_:Yb (0~100 mol%) core-shell nanoparticles under 808 nm excitation.


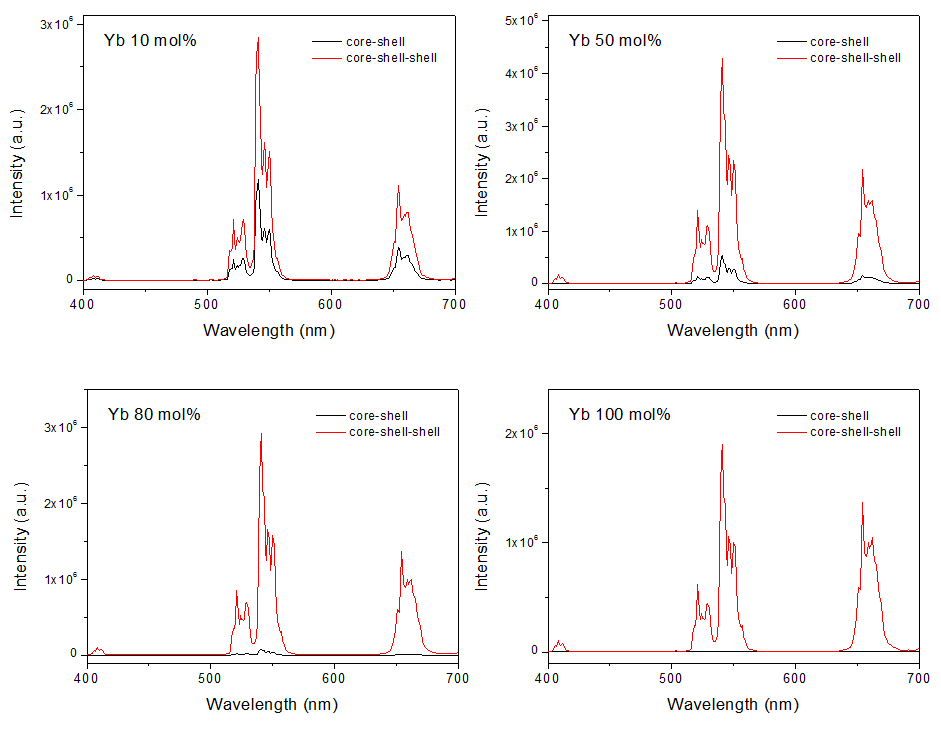


**Figure S6.** A comparison of upconversion emission spectra from NaYF_4_:Yb/Er(20/2 mol%)@NaNdF_4_:Yb(0~100 mol%) core-shell and NaYF_4_:Yb/Er(20/2 mol%)@NaNdF_4_:Yb(0~100 mol%)@NaYF_4_:Nd(50 mol%) core-shell-shell nanoparticles under 808 nm excitation.


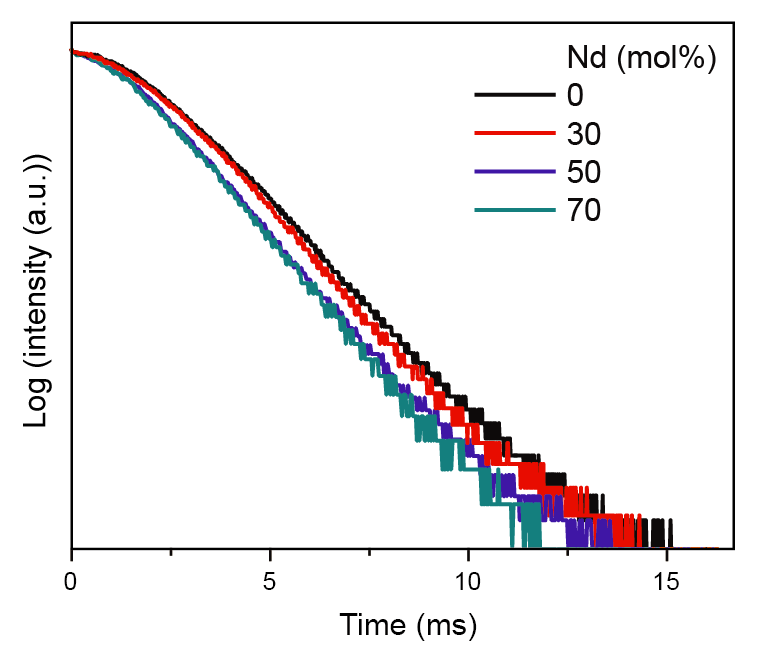


**Figure S7.** Decay curves of Er^3+^ at its ^4^S_3/2_ state monitored at 540 nm for the NaYF_4_:Yb/Er(20/2 mol%)@NaNdF_4_:Yb(50 mol%)@NaYF_4_:Nd(0~70 mol%) core-shell-shell nanoparticles under pulsed 808 nm excitation.


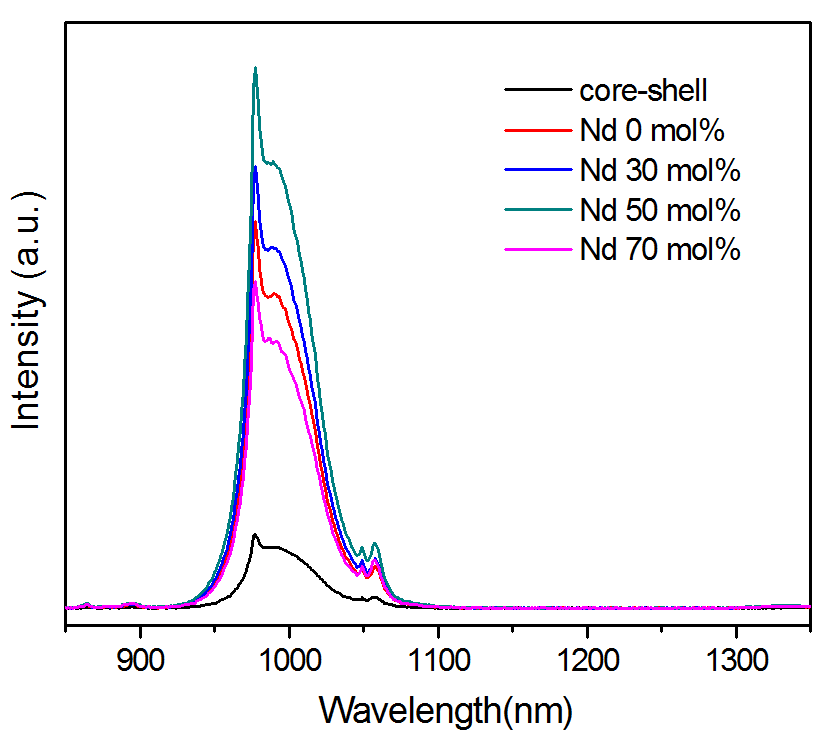


**Figure S8.** Near infrared emission spectra from NaYF_4_:Yb/Er(20/2 mol%)@NaNdF_4_:Yb(50 mol%)@NaYF_4_:Nd(50 mol%) core-shell-shell nanoparticles under 808 nm excitation. Note that the emission spectrum from the NaYF_4_:Yb/Er(20/2 mol%)@NaNdF_4_:Yb(50 mol%) core-shell-shell nanoparticles under 808 nm excitation was also plotte for comparison.
